# Transforming Growth Factor β Signaling Pathway Associated Gene Polymorphisms May Explain Lower Breast Cancer Risk in Western Indian Women

**DOI:** 10.1371/journal.pone.0021866

**Published:** 2011-08-04

**Authors:** Narendra N. Joshi, Mithila D. Kale, Sujata S. Hake, Sadhana Kannan

**Affiliations:** 1 Cancer Research Institute, Advanced Centre for Treatment, Research and Education in Cancer (ACTREC), Tata Memorial Centre (TMC), Kharghar, Navi Mumbai, India; 2 Epidemiology and Clinical Trials Unit - Clinical Research Centre, Advanced Centre for Treatment, Research and Education in Cancer (ACTREC), Tata Memorial Centre (TMC), Kharghar, Navi Mumbai, India; Ohio State University Medical Center, United States of America

## Abstract

Transforming growth factor β1 (*TGFB1*) T29C and TGF β receptor type 1 (*TGFBR1*) 6A/9A polymorphisms have been implicated in the modulation of risk for breast cancer in Caucasian women. We analyzed these polymorphisms and combinations of their genotypes, in pre menopausal breast cancer patients (N = 182) and healthy women (N = 236) from western India as well as in breast cancer patients and healthy women from the Parsi community (N = 48 & 171, respectively). Western Indian women were characterized by a higher frequency of TGFB1*C allele of the TGF β T29C polymorphism (0.48 *vs* 0.44) and a significantly lower frequency of TGFBR1*6A allele of the *TGFBR1* 6A/9A polymorphism (0.02 *vs* 0.068, p<0.01) as compared to healthy Parsi women. A strong protective effect of TGFB1*29C allele was seen in younger western Indian women (<40 yrs; OR = 0.45, 95% CI 0.25–0.81). Compared to healthy women, the strikingly higher frequencies of low or intermediate TGF β signalers in patients suggested a strong influence of the combination of these genotypes on the risk for breast cancer in Parsi women (for intermediate signalers, OR = 4.47 95%CI 1.01–19.69). The frequency of low signalers in Parsi healthy women, while comparable to that reported in Europeans and Americans, was three times higher than that in healthy women from western India (10.6% *vs* 3.3%, p<0.01). These observations, in conjunction with the low incidence rate of breast cancer in Indian women compared to White women, raise a possibility that the higher frequency of TGFB1*29C allele and lower frequency of TGFBR1*6A allele may represent important genetic determinants that together contribute to a lower risk of breast cancer in western Indian women.

## Introduction

Germline mutations in various cancer susceptibility genes can account for only around 10% of all breast cancer cases [1]. Thus, the genetic basis of breast cancer in the majority of patients who do not have a family history for malignant disorders remains poorly understood. The influence of common genetic variants on the risk for breast cancer has been suggested by many studies [2], [3]. The identification of a number of such variants in recent years highlights the interest and efforts in this direction [4], [5]. These efforts could aid in population based screening to identify high risk subjects [6]. In this context, the naturally occurring functional polymorphisms in TGF β1 and TGF β Receptor1 genes have been extensively studied for their influence on the risk for various malignant disorders [4], [7]–[9]. The effect of modulation of TGF β1 expression on the growth of mammary tumors in murine models [10], [11], contribution of mutations in genes coding for members of the TGF β signaling pathway to the development or progression of various cancers [12], [13], and the association of higher levels of TGF β1 in tumor tissues with lymph node metastases as well as poor prognosis [14], [15], all provide a strong rationale for such studies.

In breast cancer patients, *TGFB1* T29C (L10P; rs1800470) and *TGFBR1* 6A/9A remain the two most extensively studied polymorphisms. In *TGFB1* T29C polymorphism, a replacement of Leucine by Proline at position 29 (*TGFB1 codon10* T>C) has been shown to result in an increased secretion of the cytokine [16]. A nine base pair deletion in the repeat sequence of exon 1 of the *TGFBR1* gene, giving rise to the TGFBR1*6A allele [17], was found to result in weaker cytokine induced response compared to the wild type, TGFBR1*9A allele [18]. The hypomorphic behavior of this allele was also reflected in its association with weaker radiation induced response of lymphocytes [19]. Moreover, the allele has been implicated in TGF β independent enhancement of migration and invasion of MCF-7 cells [20].

With regard to the risk for breast cancer, it has been proposed that the presence of TGFB129*C allele that is associated with higher production of the cytokine, would provide protection in the initial stages by virtue of its anti-proliferative action on mammary epithelial cells but would enhance the risk at later stages by promoting invasion as well as spread of the disease [21]. Findings of the Breast Cancer Association Consortium, the only large scale study that has clearly addressed this possibility, favor such a hypothesis and place those with TGFB1*CC genotype at a higher risk of the disease [4]. Similar observations have been reported in studies on European women [16]. Two recent meta analyses of studies on population based controls [7], [8] also arrived at the same conclusion. On the other hand, some studies have failed to detect any association [22]–[25] or have reported a weak protective influence of TGFB1*CC genotype in older American women and pre menopausal Japanese women [26], [27]. Probable age and ethnicity related variations within and across the study populations are thought to be responsible for the discordant data. Similar considerations may also explain the discordance between the findings of various studies examining the influence of *TGBR1* 6A/9A polymorphism on the risk for breast cancer [18], [24], [25], [28], [29]. However, a recent meta analysis supported the increased breast cancer risk in women positive for the TGFBR1*6A allele [9]. The trend of disagreement between observations from different studies also extends to the study of the influence of the combined effect of genotypes of these polymorphisms on the risk for breast cancer. The larger impact of combinations of genotypes that result in weak cytokine signaling leading to increased risk for the disease as observed by Kaklamani *et al* [30], was not detected in two other studies [24], [25] suggesting a need for additional studies with larger cohorts.

Population based data regarding these polymorphisms in Indian women remains limited [31], [32]. The increasing incidence of breast cancer in the Indian subcontinent, especially in the younger age group [33], emphasizes the need for studies addressing the genetic basis of risk for breast cancer in the population. Further, over forty percent of breast cancer patients in India are diagnosed at age ≤45 years as against fifteen percent patients in western countries, an observation supported by the trends reported in a recent study [34]. We therefore examined the influence of TGF β1 T29C and *TGFBR1* 6A/9A gene polymorphisms in pre menopausal women from western India. To that effect the study was restricted to Marathi-speaking (Maharashtrian) subjects from Western India, living within 200 km radius around the state-capital city of Mumbai (who constitute 80% of the population of the state of Maharashtra). In parallel, we have studied these polymorphisms in breast cancer patients and healthy women from the Parsi (Zoroastrian) community. Parsis in India represent a geo-ethnically isolated community [35]. The advantages of genetic studies in isolated populations have long been recognized [36]. Further, breast cancer is the most common cancer in Parsi women with a 1.5 fold higher age adjusted incidence rate compared to the rate in non-Parsi Indian women [37]. In view of these considerations, it was of relevance to carry out a parallel study in subjects from these two communities.

The comparison of genotypes in healthy subjects from the two ethnically distinct communities studied, and the comparisons between patients and healthy subjects within each of the groups have revealed novel trends with significant implications. Further, in terms of the influence of high TGF beta1 producing genotype (TGFB1*CC) on the risk for breast cancer, our results reveal trends that are opposite to those reported in Caucasian women. The basis for these observations and their implications has been discussed with due supporting literature.

To our knowledge, this is the first study of *TGFBR1* 6A/9A genotypes in an Indian cohort. Similarly, this study also represents the first report of combined analysis of these two polymorphisms in populations outside the western hemisphere.

## Materials and Methods

### Study Subjects

#### Ethics Statement

This case-control study was carried out with the approval of the ethics committee of the Tata Memorial Hospital, Mumbai. The participants were recruited after obtaining their informed, written consent.

Reproductive health history was collected with an appropriately designed questionnaire. One hundred and eighty two Maharashtrian (Marathi-speaking, Hindu) pre menopausal women with confirmed diagnosis of breast cancer treated at the Tata Memorial Hospital during 1999–2005 were recruited within twelve to eighteen months of diagnosis. Lack of family history for malignant disorders could be confirmed for 75% of the cases. Two hundred and thirty six unrelated, healthy premenopausal Maharashtrian women without family history for cancer were recruited from the community. Parsi women with breast cancer (N = 48) comprised of those treated in various hospitals in the city. Mean post diagnosis duration for these patients was 4.8±4.2 yrs (Median  = 3), with sixty two percent of the patients recruited within five years of diagnosis. Healthy, unrelated Parsi women (N = 171) were recruited from the community. Eight Parsi patients as well as ten healthy women volunteers reported a strong family history for cancer.

The mean age was lower in healthy Maharashtrian controls than in patients (33.1±7.5 and 39.1±6.6, respectively; p<0.01). The available data indicated comparable age at menarche and age at first child birth in the two groups (Table 1). In Parsi subjects, the mean age of the control group was 57.1±9.2 yrs while that of patients was 50.3±9 yrs (p<0.01). The age at menarche, first child birth and menopause was similar in the two groups (Table 1).

**Table 1:** Reproductive health history of the study subjects.

	Maharashtrian Subjects*ab		Parsi Subjects$c-e	
	Controls	Patients	Controls	Patients
	N = 236	N = 182	N = 171	N = 48
Age	33.1±7.5^#^	39.1±6.6	57.1±9.2	50.3±9
Age at Menarche	13.9±1.3 (190)^ ^^	14.7±3.5 (115)	12.7±1.6 (147)	12.9±1.7 (42)
Age at first child – birth	24.1±4.7 (136)	22.7±3.7 (90)	26.7±3.9 (125)	26.5±3.9 (36)
Age at Menopause	-	-	46.6±5.0 (131)	46.4±3.4 (29)

# (Mean ± SD); ^ Numbers in parentheses represent the number of subjects with available information. For patients, age represents age at diagnosis. *a) Fifty three healthy subjects and six patients were unmarried; b) A number of young healthy subjects were recently married and hence nulliparous. $ c) Twenty healthy subjects and ten patients were premenopausal; d) Sixteen healthy subjects and six patients were unmarried; e) Twenty six healthy women and five patients were nulliparous.

### Genotyping

Isolation of DNA for genotyping was carried out as described in our earlier report [38]. Genotyping for *TGFB1* T29C polymorphism was performed by PCR–SSP based method, using primers described by Perrey *et al* [39]. Products were analyzed on 1.5% agarose gel stained with ethidium bromide. DNA samples of known genotypes from the *International Histocompatibility Workshop Group* (IHWG) reference panel were used to validate the PCR conditions and as controls in each experiment. Results were further confirmed by DNA sequencing in representative samples. Genomic DNA was amplified using primers, GAGGCCCTCCTACCTTTTG (F) and GCAGCTTGGACAGGATCT (R) as described in the SNP500 cancer database http://snp500cancer.nci.nih.gov/sequencing_assays.cfm?snp_id=TGFB1-01. The amplified products were sequenced by the standard method using big dye terminator kit (ABI) on 3100AVANT Genetic Analyzer (Applied Biosystems).

For *TGFBR1* 6A/9A genotyping, exon 1 of *TGFBR1* was amplified using primers described by Kaklamani *et al* [30]. Ten microlitres reaction mix contained 50 ng of DNA, 1X PCR buffer (Fermentas), 0.2 mM dNTPs, 1.5 mM Mg^2+^, 10% DMSO, 0.1% BSA and 0.5 units of Taq polymerase (Fermentas) along with 1.0 µM primers. PCR conditions were as follows −95°C for 5 min, 35 cycles of 94°C for 30 s, 65°C for 30 s, 72°C for 30 s, followed by extension at 72°C for 5 min. PCR products (256/247 bp) were analyzed on 2% agarose gel stained with ethidium bromide. The genotypes were also confirmed by running the products on 10% PAGE and by sequencing of representative samples.

### Statistical analysis

Chi-square test was used to find out if the genotype frequencies in controls are in Hardy-Weinberg equilibrium and also to determine the significance of difference in genotype frequencies between different groups. For various comparisons, two sided p values were calculated using Fisher’s exact test. To estimate the risk for breast cancer, the frequencies of subjects homozygous for the wild type alleles, namely TGFB1*29T and TGFBR1*9A, were taken as reference for the respective polymorphisms. For Maharashtrian subjects, the risk was determined after adjusting for age, and was expressed as odds ratio with 95% confidence interval. In each case, the odds ratios were computed for dominant, additive and recessive models. Further, various combinations of genotypes of these two polymorphisms were categorized into high, intermediate and low signalers [30], wherein subjects with TGFB1*CC and TGFBR1*9A/9A genotype combination were identified as high signalers in accordance with the high TGF β levels and strong TGFBR1 activity associated with these genotypes. Individuals homozygous for the allele associated with either higher TGF β levels or with strong signaling (TGFB1*CC or TGFBR1*9A/9A) were grouped as intermediate signalers, and subjects with the remaining combinations constituted low signalers. The risk was estimated for the latter two groups with high signalers as reference. SPSS software (version 15.0) was used for statistical analysis. Power calculations were performed using Quanto [40].

## Results

Two hundred and twenty four healthy controls (95%) and one hundred and sixty (88%) premenopausal breast cancer patients from the Maharashtrian community could be genotyped for the two polymorphisms studied. Similarly, one hundred and sixty (94%) healthy Parsis and forty three Parsi patients (95%) could be genotyped and included in the analysis. The genotype frequencies for the two polymorphisms studied were in agreement with Hardy-Weinberg equilibrium in healthy subjects from both the communities.

### Association of TGF β1 T29C polymorphism with risk for breast cancer

The frequency of the variant allele (TGFB1*29C) in healthy Maharashtrian women was higher compared to that in healthy Parsi women (0.48 vs 0.44; Table 2). The differences in genotype frequencies between healthy Maharashtrian and Parsi women were not statistically significant. In Parsis, genotype frequencies differed significantly between healthy subjects and patients (p = 0.002; Table 2). The higher frequency of TGFB1*TC genotype in Parsi patients was further confirmed by sequencing 50% of the randomly selected samples. The genotype frequencies were also analyzed in different subgroups of Maharashtrian patients, based on age at diagnosis (< or ≥40 yrs).

**Table 2:** Frequencies of *TGFB1*T29C and *TGFBR1* 6A/9A genotypes in the study populations.

		Maharashtrian Subjects		Parsi Subjects	
		Controls	Cases	Controls	Cases
***TGFB1*** ** T29 C**		**N (%)**	**N (%)**	**N (%)**	**N (%)**
	TT	61 (27.2)	58 (36.2)	53 (33.1)	9 (20.9)*
	TC	109 (48.7)	72 (45.0)	72 (45.0)	32 (74.4)
	CC	54 (24.1)	30 (18.8)	35 (21.9)	2 (4.7)
	T%	51.6	58.8	55.6	58
	C%	48.4	41.2	44.4	42
***TGFBR1*** ** 6A/9A**		**N (%)**	**N (%)**	**N (%)**	**N (%)**
	9A	213 (95.9)	163 (97.6)	148 (87.6)	33 (78.6)
	9A6A	9 (4.1)	4 (2.4)	19 (11.2)	8 (19.0)
	6A	0	0	2 (1.2)	1 (2.4)
	9A%	98	98.8	93.2	88.1
	6A%	2**	1.2	6.8	11.9

*p<0.01 for *TGFB1* T29C genotype frequencies in Parsi healthy controls *vs* patients.

**p<0.01 for TGFBR1*6A allele frequency in healthy Maharashtrian *vs* Parsi women.

A trend towards a protective effect of TGFB129*C allele (OR = 0.66; CI, 0.42–1.02) was noted in Maharashtrian subjects for the dominant model only (Table 3). In Parsi subjects, the increased risk suggested in carriers of TGFB129*C allele (dominant model) and a protective influence of the TGFB1*CC genotype recessively, were not statistically significant (Table 3). In younger Maharashtrian subjects, (<40 yrs), stronger protection observed in carriers of the TGFB1*29C allele remained unaffected even after adjustment for age (OR = 0.50; CI, 0.27–0.94; Table 4). In the same age group, subjects homozygous for the variant allele were significantly protected compared to those homozygous for the wild type allele (additive model) but the trend was weaker for the recessive model.

**Table 3:** *TGFB1* T29C polymorphism and risk for breast cancer in Maharashtrian and Parsi women.

	Maharashtrian Subjects			Parsi Subjects	
	Cases/Controls	OR† (95% CI)	OR^‡^ (95% CI)	Cases/Controls	OR† (95% CI)
Genotype					
***TGFB1*** *T29C					
*Dominant Model*					
TT	58/61	1	1	9/53	1
TC/CC	102/163	0.66 (0.42-1.02)	0.73 (0.46-1.17)	34/107	1.87 (0.84-4.19)
***Additive Model***					
TT	58/61	1	1	9/53	1
TC	72/109	0.69 (0.44-1.11)	0.75 (0.46-1.24)	32/72	2.62 (1.15-5.94)
CC	30/54	0.58 (0.33-1.04)	0.68 (0.37-1.26)	2/35	0.34 (0.07-1.65)
***Recessive Model***					
TT/TC	130/170	1	1	41/125	1
CC	30/54	0.73 (0.44-1.2)	0.8 (0.47-1.38)	2/35	0.17 (0.04-0.76)*

† Crude ORs; ^‡^ Age adjusted ORs; *p<0.05.

**Table 4:** *TGFB1* T29C gene polymorphism and risk for breast cancer in Maharashtrian subjects with respect to the age of onset.

	<40 yrs			≥40 yrs		
	Cases/Controls	OR† (95% CI)	OR‡ (95% CI)	Cases/Controls	OR† (95% CI)	OR‡ (95% CI)
Genotype						
***TGFB1*** **T29C**						
***Dominant Model***						
TT	29/39	1	1	29/22	1	1
TC/CC	41/123	0.45 (0.25-0.81)**	0.50 (0.27-0.94)*	61/40	1.16 (0.58-2.29)	1.17 (0.59-2.32)
***Additive Model***						
TT	29/39	1	1	29/22	1	1
TC	30/79	0.51 (0.27-0.97)*	0.56 (0.28-1.11)	42/30	1.06 (0.51-2.19)	1.08 (0.52-2.23)
CC	11/44	0.34 (0.15-0.76)**	0.39 (0.16-0.92)*	19/10	1.44 (0.56-3.71)	1.44 (0.56-3.71)
***Recessive Model***						
TT/TC	59/118	1	1	71/52	1	1
CC	11/44	0.5 (0.24-1.04)	0.55 (0.25-1.18)	19/10	1.39 (0.59-3.24)	1.38 (0.59-3.22)

† Crude ORs;

‡ Age adjusted ORs;

*p<0.05,

**p<0.01.

### *TGFBR1* 6A/9A polymorphism and risk for breast cancer

table-2-captionThe distribution of genotype frequencies for the *TGFBR1* 6A/9A polymorphism in Maharashtrian healthy women was significantly different from that in Parsi healthy women (p = 0.006; Table 2). Maharashtrian patients and controls were characterized by the absence of TGFBR1*6A homozygous individuals and comparable frequencies of *TGFBR1* 6A/9A genotypes. Risk assessment based on the observed genotype frequencies was inconclusive in Parsis as well as Maharashtrians (Table 5).

**Table 5:** *TGFBR1* 6A/9A polymorphism and risk for breast cancer in Maharashtrian and Parsi women.

	Maharashtrian Subjects			Parsi Subjects	
	Cases/Controls	OR† (95% CI)	OR‡ (95% CI)	Cases/Controls	OR† (95% CI)
Genotype					
***TGFBR1*** **6A9A**					
***Dominant Model***					
9A9A	163/213	1	1	33/148	1
9A9A/9A6A	4/9	0.58 (0.18-1.92)	0.83 (0.23-3.0)	9/21	1.92 (0.81-4.57)
***Additive Model***					
9A9A	163/213	1	1	33/148	1
9A6A	4/9	0.58 (0.18-1.92)	0.83 (0.23-3.0)	8/19	1.89 (0.76-4.68)
6A6A	0/0	-	-	1/2	2.24 (0.19-25.47)
***Recessive Model***					
9A9A/9A6A	167/222	1	1	41/167	1
6A6A	0/0	-	-	1/2	2.04 (0.18-23.01)

† Crude ORs;

‡ Age adjusted ORs.

### TGF β1 signaling strength in relation to the risk for breast cancer

Analysis of combinations of the *TGFB1* T29C and *TGFBR1* 6A/9A genotypes revealed significantly elevated frequency of low signalers in healthy Parsi women compared to their Maharashtrian counterparts (10.6% *vs* 3.2%; p<0.01; Table 6). The suggested increased risk for the disease in intermediate signalers among Maharashtrian women was not statistically significant (additive model, Table 6). In Parsi women, the intermediate and low signaling status conferred significantly higher risk for breast cancer with OR of 4.47 (CI, 1.01–19.69; p = 0.05) and 8.47 (CI 1.64–43.72; p = 0.01), respectively (Table 6). Higher risk for intermediate and low signalers was also seen for dominant as well as recessive model but was statistically significant in the former case only.

**Table 6:** Analysis of association between predicted̂ TGF β signaling strength and risk for breast cancer.

	Maharashtrian Subjects			Parsi Subjects	
	Cases/Controls	OR† (95% CI)	OR‡ (95% CI)	Cases/Controls	OR† (95% CI)
Predicted Signaling Status					
***Dominant Model***					
HS	29/52	1	1	2/32	1
IS + LS	128/167	1.37 (0.83-2.29)	1.27 (0.74-2.2)	40/128	5 (1.15-21.79)*
***Additive Model***					
HS	29/52	1	1	2/32	1
IS	126/160	1.41 (0.85-2.35)	1.29 (0.75-2.24)	31/111	4.47 (1.01-19.69)*
LS	2/7	0.51 (0.1-2.63)	0.66 (0.12-3.71)	9/17	8.47 (1.64-43.72)*
***Recessive Model***					
HS + IS	155/212	1	1	33/143	1
LS	2/7	0.39 (0.08-1.91)	0.54 (0.10-2.87)	9/17	2.29 (0.94-5.59)

† Crude ORs;

‡ Age adjusted ORs;

*p≤0.05;

**p≤0.01.

HS - High Signalers: CC/9A9A; IS - Intermediate Signalers: TT/9A9A, CC/9A6A, CC/6A6A or TC/9A9A;

LS - Low Signalers: TT/6A6A, TT/9A6A, TC/9A6A or TC/6A6A ^(Ref. 30).

## Discussion

In Maharashtrian subjects, the observed frequencies for *TGFB1* T29C polymorphism were comparable to those reported in North Indian subjects [31]. Together these data reveal relatively higher frequency of TGFB1*29C allele in western and northern Indians, as compared to those of European descent [4], [7], [8]. Conversely, another study in healthy subjects from South India [32] reporting allele and genotype frequencies similar to those reported in Whites might reflect extensive variation across ethnic groups, and thus demands additional studies in diverse Indian population groups. The TGFB1*29C allele frequency in Parsi controls was comparable to that observed in their ancestral neighbors, the Iranian controls (47% and 43%) as reported by two groups [41], [42].

The strikingly lower frequency of TGFBR1*6A allele in Maharashtrian women compared to that seen in Parsis [2.0% vs 6.8%, respectively; p<0.01; *power*-67%) is also in contrast to data from Whites, where the frequency of this allele varies between six and eleven percent [10]. Two studies in Chinese subjects have reported disparate frequencies of 6.2% [43] and 11% [44] for this allele. Thus the frequency of TGFBR1*6A allele observed in Maharashtrian women in the present study is the lowest reported so far.

### Association of TGF β1 T29C and *TGFBR1* 6A/9A polymorphisms with breast cancer risk

Our findings in Maharashtrian premenopausal women suggest an association between the presence of TGFB1*29C allele (45% power), and a reduced risk for the disease especially in the younger age group (<40 yrs; 72% power). These observations are in direct contrast with those from the Breast Cancer Association Consortium [4], a multicentric study that predominantly included subjects of European descent from different continents. On the other hand, our findings concur with the observations in premenopausal Japanese women and in American women [27], [30]. The protection conferred by higher frequency of this allele associated with higher TGF β production would be compatible with its ability to suppress development of mammary tumors [10]. The possible contribution of low frequency of the TGFBR1*6A allele to the observed effect of the TGFB1*29C allele on the risk for the disease in Maharashtrian women cannot be ruled out.

An attempt was also made to analyse the association of *TGFB1* T29C genotypes with hormone receptor status in Maharashtrian patients. Of the one hundred and twenty two patients where the information was available, the hormone receptor negative tumor bearing subjects were characterized by a strikingly lower frequency of TGFB1*CC genotype compared to the hormone receptor positive group (8 of 64 *vs* 10 of 40; Table S1). Thus a trend towards an association between lower frequency of hormone receptor negative tumors and TGFB1*CC genotype was suggested (p<0.05; Fig S1). Similar findings have been reported by Kaklamani et al. [30] in a cohort that included patients from all age groups.

The associations seen in our study however must be viewed with caution in view of the inadequate power of our study (Table 3 & 4). The data from the Breast Cancer Association Consortium that comprised of nearly thirteen thousand cases and fifteen thousand controls revealed a 16% increased risk for rare homozygotes with nearly 100% power [4]. The frequency of the minor allele for TGFB1*T29C polymorphism varies from 38% to 44% in the majority of populations across the world [9]. Thus, for the dominant model, over three thousand patients and a similar number of healthy controls would have to be studied in order to detect a 15% increase in associated risk with adequate power (80% at α = 0.05) and, for the recessive model, over five thousand patients and controls would have to be studied. Similarly, for an equivalent increase in risk associated with TGFBR1*6A allele (frequency 10%) with adequate power, sixteen thousand (dominant model) and forty six thousand (recessive model) patients and controls would have to be studied [40]. Accordingly, for TGFB1*T29C polymorphism with the observed allele frequencies in the western Indian population, to detect a 15% change in the risk for breast cancer, nearly four thousand patients and an equal number of healthy controls would be needed to achieve a power of 80% (at α = 0.05). Similar computations for TGFBR1*6A allele indicate that for a fifteen percent increment in the risk for breast cancer [10] due to presence of this allele, nearly twenty thousand patients and controls would be needed whereas, to determine its effect recessively, over a million subjects would be needed. In view of these considerations, the possibility that the observed association in our study of western Indian women is a false positive result cannot be ruled out.

### TGF β1 signaling status and risk for breast cancer

**Figure 1:**
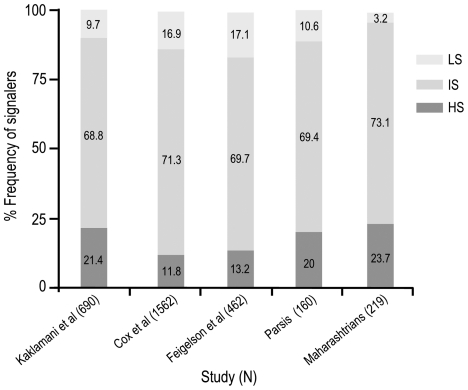
Comparison of frequencies of High, Intermediate and Low signalers in various studies reported so far. Numbers in parentheses represent the number of healthy subjects studied in each report.

An important consequence of the relatively higher frequency of TGFB1*29C allele and lower frequency of TGFBR1*6A allele in healthy Maharashtrian women was revealed when combinations of these genotypes were analyzed. Categorization of individuals into low, moderate and high TGF β1 signalers based on an approach described by Kaklamani *et al* [30], revealed strikingly lower frequency of low signalers in healthy Maharashtrian women compared to that in Parsi women (3.2% *vs* 10.6%; p<0.01). A comparison of the frequencies of high, intermediate and low signalers among healthy subjects reported in literature (Fig 1) indicates that the frequency of low signalers is strikingly reduced in women from western India.

In view of the small numbers of patients studied from the Parsi community, the observed high risk (OR >4) for breast cancer indicated for low and intermediate (p = 0.01 and p = 0.05, respectively; Table 6) signalers in these subjects warrants a note of caution. The observation may reflect the high degree of genetic homogeneity [36] and/or an ethnicity-specific effect [45]. Parsis have been known to marry within the community for over the past few centuries and may have experienced a genetic bottleneck following their arrival in Indian seven hundred years ago [35].

### Basis for the prevalence of TGF β signaling mediated inhibition in Maharashtrian women and its implications

Several lines of evidence prompt us to suggest that the distinct trends in genotype and allele frequencies of the two polymorphisms in Maharashtrians may reflect the effect of environmental forces. A higher frequency of genotypes contributing to elevated pro-inflammatory responses has been reported in populations from tropical regions [46], [47]. Commensurate with these observations, Asians are characterized by higher levels of markers associated with chronic inflammation as well as IgG1 levels, as compared to Whites [48], [49]. In view of the significant contribution of inflammation to the initiation and progression of malignancies [50], these features may also contribute to an overall earlier age of onset of malignant disorders in the region [51]. The prevalence of TGF β mediated inhibition in Maharashtrian subjects revealed in this study may have emerged as a counteracting force to the selection of the pro-inflammatory genotypes to minimize the risk of autoimmunity [52]. This in turn could also suppress the development of malignancies by virtue of its anti-proliferative effects on the mammary epithelial cells [21]. Thus, the effects of higher frequency of TGFB1*29C allele, alone and in association with the lower frequency of TGFBR1*6A allele, may have implications for the lower incidence of breast cancer in this population.

The large body of data supporting the association of *TGFB1* T29C and *TGFBR1* 6A/9A polymorphisms with the risk for breast cancer provides a strong basis to endorse the view that the combinations of genotypes resulting in weak TGF β signaling would have a greater impact on the risk for the disease - although the findings of epidemiological studies in this context remain equivocal [24], [25], [30]. Therefore, the population attributable risk associated with such genotype combinations or their contribution to the total disease burden, a function of frequency and penetrance also remains unresolved. Our results implicate TGFB1*29C genotype in modulating the risk for breast cancer in western Indian women. Further this population is characterized by a lower frequency of TGFBR1*6A allele. These observations, and the predicted influence of the combination of genotypes of the two polymorphisms studied, permit us to argue that three-fold lower frequency of low signalers may represent one of the mechanisms that would contribute to the reduced risk for breast cancer in Maharashtrian women compared to Parsis or Whites, a possibility that would be commensurate with the known difference in breast cancer incidence rates between the Indians and Whites [53].

In summary, the present study on *TGFB1* T29C and *TGFBR1* 6A/9A polymorphisms in relation to breast cancer risk has revealed important differences between the genotypes and allele frequencies in premenopausal Maharashtrian women, compared to Parsi and White women. These may have implications for the lower risk for the disease in Maharashtrian women. Available data supports the possibility of environmental influence as one of the driving forces giving rise to such an ethnicity-specific genetic variation [46], [47]. The influence of high TGF β1 producing genotype (TGFB1*CC) in western Indian women suggested by our study and in White women by others [4], despite opposite trends, points towards its considerable penetrance. The trends revealed in the present study raise a number of important possibilities and stress need for a larger study coupled with the assessment of clinical data. Such a study could also provide a valuable prognostic parameter, especially in the context of earlier age of onset and prevalence of an aggressive disease in Indian patients.

## Supporting Information

Figure S1 **Analysis of association of** ***TGFB1*** **T29C genotypes with hormone receptor status in Maharashtrian subjects.** OR – Age adjusted odds ratios with 95% CI; n = 224; **p<0.01; *p = 0.05 for TGFB1*CC genotype.
(TIF)

Table S1 **Distribution of various TGFB1 T29C genotypes with respect to ER/PR status.**
(DOC)
