# Supplementary figures and images for: Transforming Growth Factor β Signaling Pathway Associated Gene Polymorphisms May Explain Lower Breast Cancer Risk in Western Indian Women
Source: PLoS One. 2011 Aug 4;6(8):e21866. doi: 10.1371/journal.pone.0021866 (PMC3150347; doi:10.1371/journal.pone.0021866)

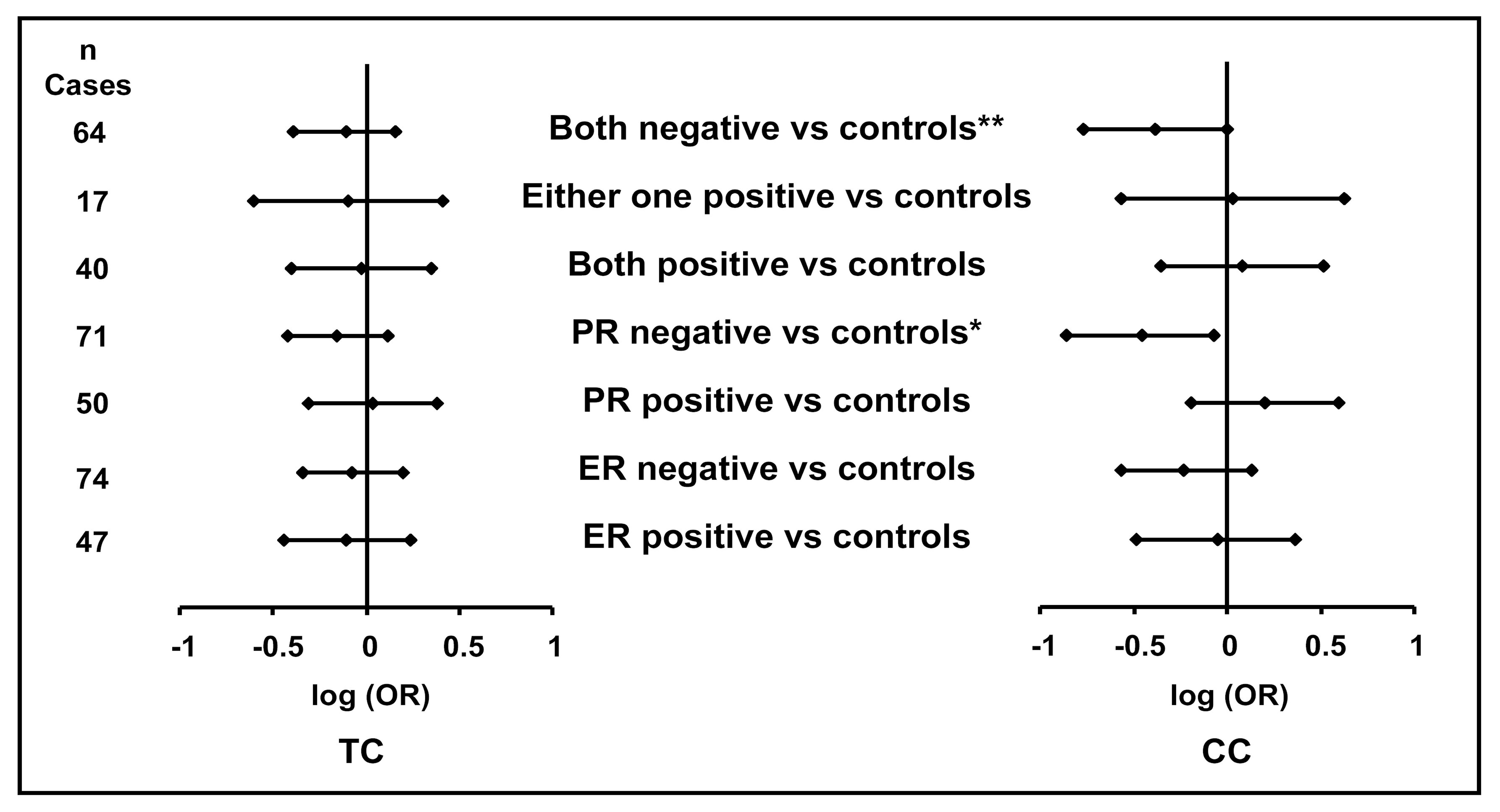

Supplement: Figure S1 — Analysis of association of TGFB1 T29C genotypes with hormone receptor status in Maharashtrian subjects. OR – Age adjusted odds ratios with 95% CI; n = 224; **p<0.01; *p = 0.05 for TGFB1*CC genotype. (TIF) [file pone.0021866.s001.tif]
