# Supplementary material for: Transforming Growth Factor β Signaling Pathway Associated Gene Polymorphisms May Explain Lower Breast Cancer Risk in Western Indian Women
Source: PLoS One. 2011 Aug 4;6(8):e21866. doi: 10.1371/journal.pone.0021866 (PMC3150347; doi:10.1371/journal.pone.0021866)
Supplement: Table S1 — Distribution of various TGFB1 T29C genotypes with respect to ER/PR status. (DOC) [file pone.0021866.s002.doc]

**Supplementary Data**

**Figure S1**

**Analysis of association of *TGFB1* T29C genotypes with hormone receptor status in Maharashtrian subjects.**

OR – Age adjusted odds ratios with 95% CI; n = 224. **p<0.01; *p=0.05 for TGFB1**CC* genotype.

| **Table S1 Distribution of various TGFB1 T29C genotypes with respect to ER/PR status** | | | | | | | | |
| --- | --- | --- | --- | --- | --- | --- | --- | --- |
| Genotype | Total Patients (%) | ER + | ER - | PR + | PR - | ER+PR+ | ER-PR- | Either + |
| TT | 44 (36) | 18 | 26 | 14 | 30 | 13 | 25 | 6 |
| CT | 55 (45) | 19 | 36 | 22 | 33 | 17 | 31 | 7 |
| CC | 23 (19) | 11 | 12 | 14 | 8 | 10 | 8 | 4 |
